# The Association Between Preoperative Malnutrition and Early Postoperative Outcomes in Children with Congenital Heart Disease: A 2-Year Retrospective Study at a Lebanese Tertiary Medical Center

**DOI:** 10.3390/children12060705

**Published:** 2025-05-29

**Authors:** Jana Assy, Christelle Yaacoub, Sarah Khafaja, Mariam Toufic Arabi, Issam El Rassi, Fadi Bitar, Nadine Yazbeck

**Affiliations:** 1 Department of Pediatrics, University Hospital of Liege Belgium, 4000 Liège, Belgium; jana.assy@chuliege.be; 2 Department of Pediatrics and Adolescent Medicine, American University of Beirut Medical Center, Beirut 1107, Lebanon; cgy02@mail.aub.edu (C.Y.); sk159@aub.edu.lb (S.K.); ma81@aub.edu.lb (M.T.A.); fbitar@aub.edu.lb (F.B.); 3 Al Jalila Children’s Specialty Hospital, Dubai 7662, United Arab Emirates; issam.elrassi@dubaihealth.ae

**Keywords:** malnutrition, wasting, stunting, underweight, congenital heart diseases, postoperative outcomes, infants and children

## Abstract

Objectives: This study aimed to describe the prevalence of malnutrition and its impact on postoperative outcomes in infants and children with congenital heart diseases (CHDs) undergoing cardiac surgeries. Methods: We conducted a single-center, retrospective review of medical records of children aged 1 month to 5 years with CHDs who underwent cardiac surgery at the American University of Beirut Medical Center (AUBMC) between January 2015 and January 2017. Anthropometric data were collected and z-scores for weight-for-age (WAZ), height-for-age (HAZ), weight-for-height (WHZ), and BMI-for-age were calculated. Malnutrition was classified based on the World Health Organization (WHO) definitions and the American Society for Parenteral and Enteral Nutrition (ASPEN) criteria. The postoperative outcomes were analyzed using bivariate and multivariable models. Results: The prevalence of malnutrition was 33.8%, with children younger than 24 months having significantly higher odds of malnutrition. The most common CHDs were ventricular septal defect (VSD) and tetralogy of Fallot (TOF), with malnutrition being significantly more prevalent in the children with VSD. Malnutrition was significantly associated with a prolonged pediatric intensive care unit (PICU) stay, with underweight being the strongest predictor. Malnourished children also had a significantly longer mechanical ventilation time (median 9.0 vs. 5.0 h, *p* = 0.017). Lower weight-for-age (WAZ) and BMI-for-age z-scores were associated with longer hospital stay, PICU stay, and mechanical ventilation durations. Conclusions: Malnutrition is prevalent among children with CHDs and is independently associated with longer hospital and PICU stays, as well as extended mechanical ventilation. Early nutritional assessment and intervention may improve postoperative outcomes in this vulnerable population.

## 1. Introduction

Congenital heart disease (CHD) is one of the most common birth defects, representing approximately one-third of all congenital anomalies. Ventricular septal defect (VSD) is the most prevalent subtype of CHD, comprising approximately 30% of the CHD cases [1]. According to the Global Burden of Diseases 2017 report, the global prevalence of CHD is estimated at 1.8 cases per 100 live births, reflecting a 4.2% increase since 1990 [1]. Moreover, CHD ranked as the seventh leading cause of infant mortality across all socio-demographic index quintiles [2]. The epidemiology of CHD varies across different ethnic populations such as Caucasian, Asian and other groups, as demonstrated in several studies [1,3,4,5]. However, data on the incidence of CHD by ethnicity in the Middle Eastern population remains limited. A study conducted in Northern Iran, published in 2014, showed that the incidence and pattern of CHD varied according to ethnicity [6].

Medical technology advancements have markedly improved the survival rates of children with CHDs; therefore, understanding the comorbidities and potential disabilities affecting this specific population is of crucial importance [7]. Notably, malnutrition remains highly prevalent among children with CHDs [8], with a multifactorial etiology that includes a hypermetabolic state and decreased energy intake, often attributed to difficulty feeding, malabsorption, and inadequate nutrient use [8]. According to the American Society for Parenteral and Enteral Nutrition (ASPEN), pediatric malnutrition was defined as “imbalance between nutrient requirement and intake, resulting in cumulative deficits of energy, protein, or micronutrients that may negatively affect growth, development, and other relevant outcomes” [9]. The World Health Organization (WHO) defines malnutrition as a broad term encompassing both undernutrition and overnutrition [10]. However, in this article, the term pediatric malnutrition will be used interchangeably for undernutrition.

Several studies have investigated the associations between nutritional status at the time of CHD surgery and postoperative outcomes, reporting heterogeneous and conflicting findings. This could be related to differences in age range, cardiac defects, underlying comorbidities, and genetic syndromes, as well as the definitions used to classify nutritional status based on anthropometric and biochemical parameters [11,12,13,14,15]. Nevertheless, preoperative malnutrition was mainly associated with mortality, infections, prolonged pediatric intensive care unit (PICU) stay, longer length of hospital stay (LOS), and increased postoperative complications [16,17]. Given that nutritional status is a potentially modifiable risk factor, early nutritional screening from diagnosis can facilitate timely nutritional intervention and ultimately optimize patient outcomes [16].

The prevalence of preoperative underweight status, stunting, and wasting varies by geographic region [8]. However, published data on malnutrition among children with CHD in low- and middle-income countries (LMICs), which may have more limited medical, financial, and social resources, are scarce, underscoring the need for further research in these settings. The aim of this study was to determine the prevalence of preoperative malnutrition among children with CHD undergoing pediatric cardiac surgery at the American University of Beirut Medical Center in Lebanon (AUBMC) and to assess its impact on postoperative outcomes, including the length of hospital and PICU stay, infections, and mortality.

## 2. Materials and Methods

### 2.1. Study Design

This study was a single-center, retrospective review of medical records of children with congenital heart diseases aged between 1 month and 5 years, who underwent cardiac surgeries between January 2015 and January 2017, at the AUBMC, a tertiary care center located in Beirut, Lebanon, serving as a referral center for pediatric patients with CHD. AUBMC hosts the Children’s Heart Center, a major center caring for children with CHDs in Lebanon. This study was approved by the institutional review board (IRB) at AUBMC (*IRB Protocol Number: PED.NY.06*). Subjects were identified through the medical record department using specific ICD-10 codes for congenital malformations of the circulatory system.

### 2.2. Inclusion and Exclusion Criteria

Infants and children aged 1 month to 5 years with confirmed diagnosis of CHD who were admitted to the pediatric units at AUBMC for surgical repair between January 2015 and January 2017 were included in the study. Patients were included in this study if data on both length (or height for children ≥ 2 years of age) and weight, as well as postoperative outcomes, were recorded.

**Figure 1:**
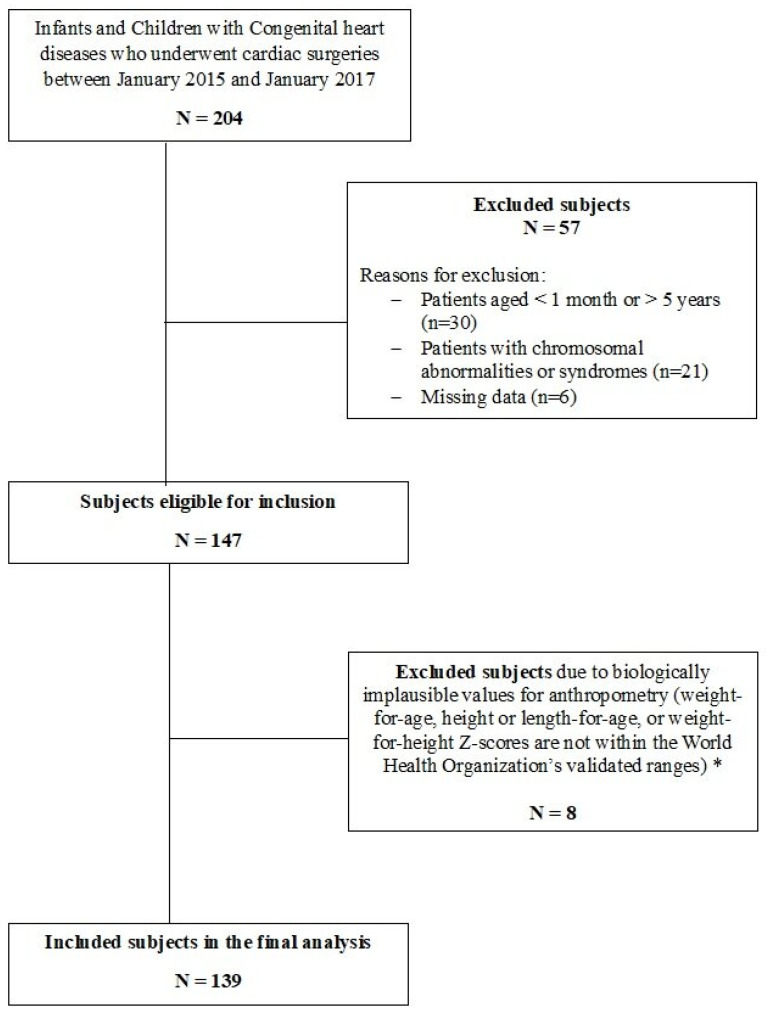
Flow diagram of the study design. * Biologically implausible values (BIVs) were defined, according to World Health Organization (WHO) Multicenter Growth Reference Study Group 2006, as height-for-age z-score (HAZ) below –6 SD or above +6 SD, weight-for-height z-score (WHZ) below –5 SD or above +5 SD, and weight-for-age z-score (WAZ) below –6 SD or above +5 SD.

Patients with chromosomal abnormalities or syndromes including Trisomy 21 and DiGeorge syndrome were excluded due to the potential confounding effect of these conditions on nutritional status. In addition, patients with biologically implausible values (BIVs) for anthropometry or missing postoperative data were excluded from the analysis (Figure 1).

Table 1 further explores demographic and clinical characteristics based on nutritional status categorized according to ASPEN criteria. Children younger than 24 months of age had 4.4 times higher odds (95% CI: 1.8–10.8) of being malnourished than those 24 months or older. There were no notable statistical differences in gender distribution, CHD type (cyanotic or acyanotic), RACHS-1 risk categories, or pulmonary hypertension between children with normal nutritional status and those who were malnourished.

### 2.3. Data Collection and Anthropometric Measurements

Data were collected using a case report form, which included the following information: basic demographic characteristics (age at the time of surgery and gender), type of CHD, presence of cyanosis, surgical complexity, and presence of pulmonary hypertension. In addition, data on perioperative management and postoperative outcomes were recorded, including LOS, duration of stay in the PICU, duration of mechanical ventilation, occurrence of infection, inotrope use, and in-hospital mortality.

At AUBMC, all pediatric patients scheduled for cardiac surgery are evaluated by a clinical nutrition team during their pre- and postoperative hospital stay. This evaluation typically occurs on an as-needed basis. Nutritional assessments focus on anthropometric measurements, caloric intake, and feeding tolerance, preoperative anthropometric measurements including weight, length (for children younger than 24 months), height (for children aged 24 months or older), and body mass index (BMI) for children aged 24 months or older were also obtained. These measurements documented in the patients’ records were taken by a trained pediatric nurse. For children below the age of two years, weight was measured using a regularly calibrated infant scale with only a clean diaper on, while length was measured in the supine position using a rigid pediatric height rod. For children aged 2 to 5 years, weight was taken using a regularly calibrated scale with the child standing barefoot, while height was measured standing on a stadiometer. BMI was calculated as the weight in kg divided by height in meters (m) squared.

To compare the anthropometric data among children, the WHO recommends the use of z-scores, where a z-score of 1 represents 1 standard deviation (SD) from the reference median. The child’s height and weight were compared to reference curves of length-for-age or height-for-age, weight-for-age, weight-for-height or weight-for-length, and BMI-for-age. For children younger than 24 months of age, the 2006 World Health Organization (WHO) Growth Standards were used, as recommended by the American Academy of Pediatrics and the Centers for Disease Control and Prevention (CDC) [18,19]. For children aged 2 years or older, the CDC growth charts were used.

Weight-for-age z-scores (WAZ), height-for-age z-scores (HAZ), and weight for length/height z-scores (WHZ) were calculated using the Peditools 20 calculator according to WHO growth charts for children aged 1 month to less than 24 months and the CDC growth charts for children aged 2 to 5 years [20]. For children aged 24 months of age or older, BMI-for-age z-scores were also calculated. It is important to note that the term length-for age z-score (LAZ) and weight-for-length (WLZ) are usually used in children younger than 24 months; however, for simplicity, the term HAZ will be used throughout this article to refer to both HAZ and LAZ, and WHZ will be used to denote both WHZ and WLZ.

### 2.4. Definitions and Categorization

According to the WHO guidelines, underweight, stunting, and wasting are three indicators used to assess nutritional status [18], and were studied separately as variables in this study. A child was classified as “underweight” if WAZ was <−2 SD. Wasting was defined as WHZ < −2 SD and stunting as HAZ  <  −2 SD [18]. Z-score cutoff values of −3 were used to identify severely underweight, wasted, or stunted status.

Global pediatric malnutrition was further defined in accordance with the new consensus statement released in 2014 by the Academy of Nutrition and Dietetics and the American Society of Parenteral and Enteral Nutrition [21]. In this study, the indicators that require only a single data point for assessment were used. Malnutrition severity was stratified into mild, moderate, and severe categories based on anthropometric measurements. Mild malnutrition was defined by a WLZ ranging from −1 to −1.9 in children aged < 2 years or a BMI-for-age z-score between −1 and −1.9 in children 2 years or older. Moderate malnutrition was characterized by a WLZ ranging from −2 to −2.9 in children aged < 2 years or a BMI-for-age z-score between −2 and −2.9 in children aged 2 years or older. Severe malnutrition was defined as follows: WLZ equal to or greater than −3 for children aged < 2 years; or BMI-for-age z-score equal to or greater than −3 for children aged 2 years or older; or a HAZ z-score of −3. Throughout this article, subjects meeting the criteria for moderate or severe malnutrition were categorized as “malnourished”, whereas those who did not meet these criteria were classified as having “normal nutritional status”. Although the consensus definition also includes mid-upper arm circumference (MUAC) as an indicator, these data were not available for the subjects included in this study.

Age groups were categorized as follows: <24 months and ≥24 months.

Infection was defined based on documented clinical signs, the presence of positive cultures (blood, urine, deep tracheal aspirate, or wound), or radiological evidence suggestive of infection, such as positive chest X-ray.

Cardiac procedures or surgeries were grouped into 1 of 6 predefined risk categories according to the Risk Adjustment for Congenital Heart Surgery (RACHS-1) system [22,23]. Category 1 represents the lowest risk of in-hospital mortality while category 6 represents the highest. For subjects who underwent a combination of cardiac surgical procedures, the highest-risk procedure determined the final classification [22,23].

A mechanical ventilation duration of ≥48 h, a PICU stay of >3 days, and a hospital stay of >7 days were considered prolonged.

BIVs were defined, according to WHO Multicenter Growth Reference Study Group 2006, as HAZ below –6 SD or above +6 SD, WHZ below –5 SD or above +5 SD, and WAZ below –6 SD or above +5 SD [24,25].

### 2.5. Statistical Analysis

All statistical analyses were performed using the Statistical Package for Social Sciences (SPSS) program, version 26.0, for Windows (IBM, Armonk, NY, USA). Simple descriptive statistics were used to describe the demographic characteristics and were represented as frequencies and percentages. Continuous variables were reported as means and standard deviations (SD). Bivariate analyses assessing the risk factors for malnutrition, stunting, wasting, underweight, and early postoperative outcomes by nutritional status were analyzed by Pearson’s Chi-Square test or Fisher’s exact test (when fewer than 5 patients were in a subgroup). Continuous variables were compared between groups, using Student’s *t*-test when the data followed a normal distribution and the non-parametric Mann–Whitney U test when the data did not meet normality assumptions. Normality was evaluated through statistical tests for normality and visual inspection of histograms and boxplots. Statistical significance was considered below a type 1 error threshold (alpha level) of 0.05.

Correlation analysis was conducted using Spearman’s rank correlation to evaluate the strength and direction of the relationships between anthropometric indicators (WAZ, HAZ, WHZ, and BMI-for-age z-score) and the continuous outcome variables, including the duration of mechanical ventilation (in hours), LOS (in days), and duration of PICU stay (in days). The strength of correlation was interpreted according to standard guidelines, with values of |ρ| ≤ 0.3 indicating weak correlation, 0.3 < |ρ| ≤ 0.7 indicating moderate correlation, and |ρ| > 0.7 indicating strong correlation.

Logistic regression models adjusting for age group, gender, and RACHS-1 risk categories were used to estimate the adjusted odds ratios (aORs) and corresponding 95% confidence intervals (CIs) for the binary outcomes including the occurrence of infection, prolonged LOS, prolonged PICU stay, and inotrope use for patients with wasting, stunting, underweight or those classified as malnourished based on the ASPEN classification. Given the relatively small sample size, we limited the number of covariates in the models, and we did not incorporate other potential predictors. Logistic regression analyses were not reported for the binary outcomes of mortality and prolonged mechanical ventilation time, due to the low event frequencies, which can restrict the use of multiple predictors. Additionally, multivariable analyses stratified by age groups and disease severity were not reported to avoid biased estimates, as such stratifications could result in categorical variables with few events.

For continuous outcomes (LOS, PICU stay, and mechanical ventilation time), a generalized linear model (GLM) with a gamma distribution and a log link function was applied to assess the relationships between malnutrition (both categorical and continuous variables) and the outcomes. The GLM was selected due to the non-normal distribution of the continuous outcomes, necessitating a method to appropriately model the skewed data. The regression coefficients (B), 95% confidence intervals (CIs), and *p*-values were reported for each malnutrition indicator.

## 3. Results

### 3.1. Demographic and Clinical Characteristics

During the 2-year study period, a total of 204 infants and children with CHD who underwent cardiac surgeries were screened for inclusion, of whom 139 subjects met the inclusion criteria and were included in the final analysis (Figure 1). The demographic and clinical characteristics of these subjects are summarized in Table S1. Among the included subjects, 79 (56.6%) were males (Table 1), 92 (66.2%) were younger than 24 months, and 47 (33.8%) were 24 months of age or older. When comparing the two age groups, no statistically significant differences were observed in terms of gender distribution or the presence of cyanosis or pulmonary hypertension. The RACHS-1 risk categories were predominantly between 1 and 3 for both age groups (89.2% overall), with no significant difference in distribution (*p* = 0.536). When malnutrition was assessed following the ASPEN criteria, the percentage of malnourished children (including both moderately and severely malnourished) was 33.8% (*n* = 47). Compared to subjects who did not meet any criteria for malnutrition, moderate and severe malnutrition was more common among children younger than 24 months of age than those 24 months or older, and this was statistically significant.

**Figure 2:**
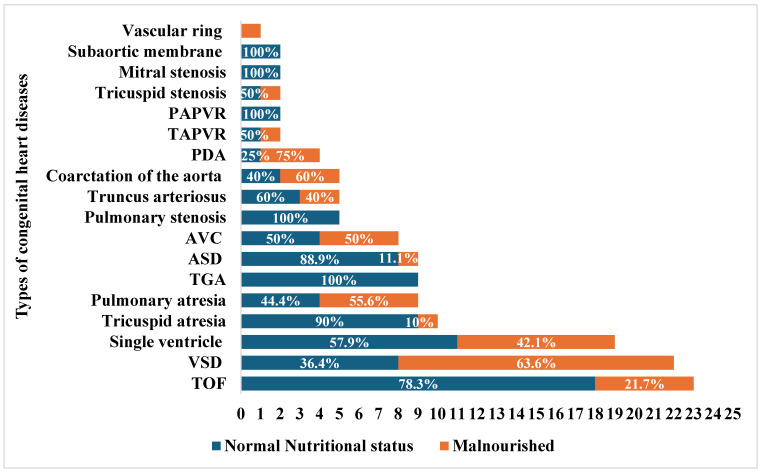
Malnutrition by congenital heart diseases. TOF: tetralogy of Fallot, VSD: ventricular septal defect, ASD: atrioventricular septal defect, GA: transposition of great arteries, AVC: atrioventricular canal defect, TAPVR: toral anomalous pulmonary venous return, PAPVR: partial anomalous pulmonary venous return. The stacked bar chart shows the count of each congenital heart disease (CHD), with blue color representing the percentage of subjects with normal nutritional status within each category of CHD, and the orange color representing the percentage of malnourished subjects.

The most common CHDs in our study were VSD (*n* = 23) and tetralogy of Fallot (TOF) (*n* = 22), followed by single ventricle (*n* = 19) and tricuspid atresia (*n* = 10). (Figure 2). The association between malnutrition and the most common types of CHDs was performed, showing that the odds of being malnourished among subjects with VSD were 6.3 times (95% CI: 1.7–23.5) greater than in those with TOF and 15.8 times (95% CI: 1.7–148.1) greater than in those with tricuspid atresia (*p*-values: 0.006 and 0.016, respectively). Compared to children with normal nutritional status, children aged younger than 24 months had 4.4 times higher odds (95% CI: 1.8–10.8) of being malnourished than those 24 months or older.

### 3.2. Anthropometric Measurements

**Figure 3:**
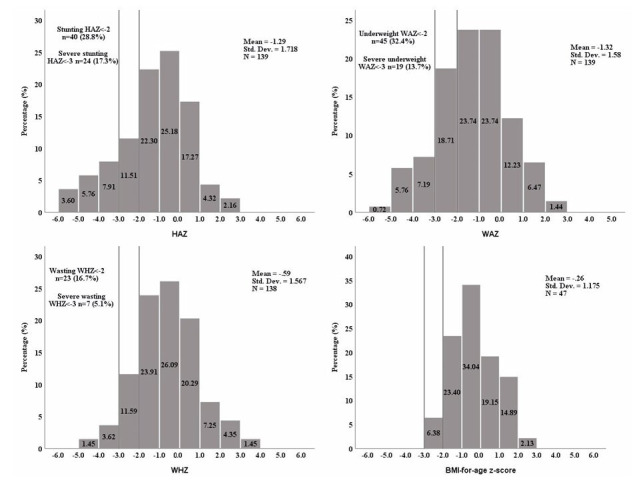
The distribution of anthropometric z-scores. HAZ: height-for-age z-score, WAZ: weight-for-age z-score, WHZ: weight-for-height z-score, BMI: body mass index (calculated only for children ≥24 months old). WHZ could not be calculated for one patient as weight-for-height charts are available only for heights between 77 and 121.5 cm.

Figure 3 illustrates the distribution of different anthropometric indicators (HAZ, WAZ, WHZ, and BMI-for age z-score) among the 139 infants and children. The mean HAZ, WAZ, and WHZ for the total study population were −1.3 (±1.7), −1.3 (±1.6), and −0.6 (±1.6), respectively. In total, 32.4% of the children aged 1–60 months were underweight (WAZ < −2), 28.8% were stunted (HAZ < −2), and 16.7% were wasted. For children aged 24 months or older (*n* = 47), the BMI-for-age z-scores were calculated, and their distribution demonstrated a less pronounced leftward skew compared to the other anthropometric parameters, with a mean z-score of −0.26 (±1.18). Statistically significant differences in HAZ and WAZ were observed between the two age groups (*p* = 0.001) (Figure S1). Among the study population, 78 children (56.1%) did not meet the criteria for any of the three malnutrition measures (stunting, wasting, or underweight), while 61 children (43.9%) met at least one measure of malnutrition, with the most common combination being underweight and stunted (*n* = 24, 17.3%), and 6 children (4.3%) met the criteria for the three measures (Figure S2).

### 3.3. Association Between Malnutrition and Early Postoperative Outcomes

#### 3.3.1. In-Hospital Mortality

Overall, in-hospital mortality was low, occurring in 2 out of 139 (1.4%) children. Mortality was documented in two subjects with normal nutritional status while none of the malnourished patients died during their hospital stay (Table 2). Given the low mortality rate, further analysis was not conducted to explore the relationship with the three indicators of malnutrition.

#### 3.3.2. Postoperative Infection

**Figure 4:**
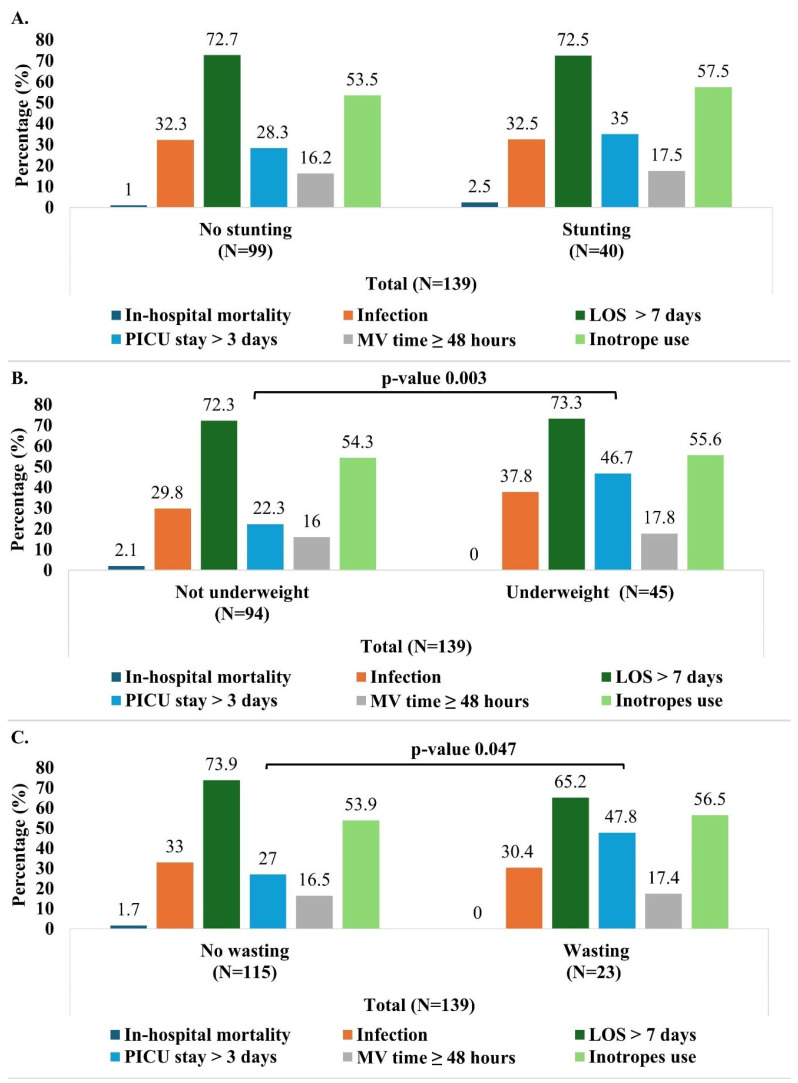
Outcomes (in-hospital mortality, infection, prolonged LOS > 7 days, PICU stay > 3 days and MV time ≥ 48 h) by malnutrition indicator (stunting (**A**), wasting (**B**), and underweight (**C**)). Pearson’s Chi-Square test was used (no expected counts less than 5). LOS: length of hospital stay; PICU: pediatric intensive care unit; MV: mechanical ventilation.

**Figure 5:**
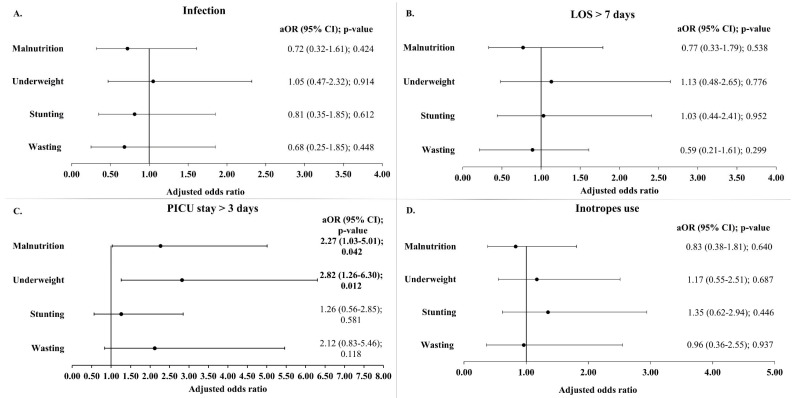
Adjusted odds ratio of infection (**A**), prolonged LOS (**B**), prolonged PICU stay (**C**), and inotrope use (**D**) by malnutrition indicator. Models were adjusted for age at time of surgery, gender, and RACHS-1 categories. LOS: length of hospital stay; PICU: pediatric intensive care unit; aOR: adjusted odds ratio; CI: confidence interval.

Analyses of postoperative infection rates did not reveal any significant association with malnutrition (Table 2 and Figure 4). Infection occurred in 31.5% of the subjects with normal nutritional status and 32.5% of the malnourished children and this difference was not statistically significant (Table 2). Also, when stratified by age groups, the infection rates did not vary between the malnourished and non-malnourished subjects (Table S2). Similarly, the infection rate did not differ between stunted versus non-stunted, wasted versus non-wasted, or underweight versus non-underweight groups (Figure 4). Likewise, after adjusting for age, gender, and RACHS-1 categories, no statistically significant relationships were found between infection rates and the different parameters of malnutrition including wasting, stunting, and underweight (Figure 5).

#### 3.3.3. LOS and Duration of PICU Stay

Overall, the mean duration of hospital stay was 12.7 (±9.6) days and the mean length of PICU stay was 4.7 (±6.7) days. Malnutrition did not significantly affect the LOS, as demonstrated in the bivariate analyses (Table 2, Tables S2 and S3, and Figure 4) and the different logistic regression models (Figure 5).

Regarding the duration of PICU stay, the median duration was longer in the malnourished children (3.0 days) compared to those with normal nutritional status (2.0 days), with this difference being statistically significant (*p*-value 0.004). Furthermore, a greater proportion of the malnourished children (44.7%) had a prolonged PICU stay (more than 3 days) compared to those with a normal nutritional status (22.8%) (*p*-value 0.008) (Table 2). These findings were consistent in children aged 24 months or older (Table S2). In the unadjusted analysis, children who were wasted or underweight were more likely to have a prolonged PICU stay compared to those not wasted or underweight (Figure 4). More specifically, children aged 24 months or older had similar trends.

In the adjusted models, as shown in Figure 5, malnutrition, in general, remained significantly associated with a prolonged PICU stay of more than 3 days (aOR 2.27 (1.03–5.01); *p*-value 0.042). Among the indicators of malnutrition, underweight was most strongly associated, increasing the odds of a prolonged stay in the PICU by 2.82 times (*p*-value 0.012). Although stunting and wasting were also associated with higher odds of prolonged PICU stay, these failed to reach statistical significance. Similarly, when the PICU stay was analyzed as a continuous variable (in days), the GLM analysis reaffirmed the association between malnutrition and underweight with a significantly longer ICU stay (Table S5).

#### 3.3.4. Mechanical Ventilation Time and INOTROPES

The malnourished children with CHD had a significantly longer mechanical ventilation time (median 9.0 h vs. 5.0 h; *p* = 0.017). However, when the mechanical ventilation time was analyzed as a categorical variable (<48 h and ≥48 h), no significant association was found between prolonged mechanical ventilation time (≥48 h) and malnutrition (Table 2). In addition, the bivariate analysis did not reveal any association between the mechanical ventilation time (both as continuous and categorial variable) and the three indicators of malnutrition (stunting, wasting, and underweight) (Figure 4 and Table S3). Finally, no statistically significant associations were found between inotrope use and malnutrition (Table 2, Figure 4 and Figure 5).

### 3.4. Secondary Analysis: Malnutrition as a Continuous Variable and Its Impact on Outcomes

Table S4 presents the correlation analysis of the anthropometric indicators and postoperative outcomes (mechanical ventilation time, LOS, and duration of PICU stay). No significant correlations were observed with the LOS and mechanical ventilation time (Table S4). However, the WAZ and HAZ showed weak but statistically significant negative correlations with the duration of PICU stay (ρ = −0.291; *p* = 0.001 and ρ = −0.229; *p* = 0.007, respectively).

In the adjusted logistic regression models, where malnutrition was assessed continuously, most of the results showed non-significant differences in the outcomes (infection occurrence, prolonged LOS of >7 days, MV time of ≥48 h, or inotrope use). However, lower WAZ and BMI-for-age z-scores were associated with a higher likelihood of prolonged PICU stay (>3 days). Specifically, a reduction of 1 SD in the WAZ and BMI-for-age z-score was associated with a 27% (*p*-value 0.021) and 71% (*p*-value 0.047) increase, respectively, in the odds of staying more than 3 days in the PICU (not represented in the tables and figures).

The results of the GLMs assessing the relationship between malnutrition and clinical outcomes are shown in Table S5. The GLM revealed that a 1SD decrease in the WAZ was significantly associated with an increase in the log of the LOS (B −0.07; 95% CI: −0.14, −0.01; *p*-value 0.036), corresponding to a 7% increase in the duration of hospital stay. Furthermore, for each 1SD decrease in the WAZ, HAZ, and BMI-for-age z-score, the log of PICU stay increased by 0.15, 0.11, and 0.21, respectively, corresponding to an approximate 16%, 12%, and 23% increase in the PICU stay (days). While most of the malnutrition indicators did not show any statistically significant association with the mechanical ventilation time, the BMI-for-age z-score revealed a negative relationship. Specifically, a reduction of 1SD in the BMI-for-age z-score was associated with a 46% increase in the mechanical ventilation time (coefficient −0.38 (−0.64, −0.13); *p*-value 0.003), suggesting that children with lower nutritional status may require extended respiratory support.

## 4. Discussion

The global burden of preoperative malnutrition in children with CHD remains a major concern [8]. Malnutrition is known to alter the immune function through disruptions of the endocrine, epithelial, and lymphoid systems, increasing the risk of postoperative infections [26,27,28]. It also leads to a reduction in muscle mass, which can compromise the respiratory function and contribute to prolonged mechanical ventilation. These factors result in longer recovery times, poor wound healing, postoperative complications, and increased mortality in this vulnerable population [26,27,28]. Published data on malnutrition in children with CHD in LMICs are, so far, scarce [29]. Therefore, our study aimed to provide important insights into the prevalence of malnutrition and its impact on postoperative outcomes in infants and children with CHDs undergoing cardiac surgeries in a tertiary care center in Lebanon.

In our retrospective study, 32.4% of the children aged 1–60 months with CHDs were underweight, 28.8% were stunted, and 16.7% were wasted, emphasizing the burden of malnutrition in this high-risk population. These findings are consistent with global trends observed in pediatric CHD populations. A large systematic review and meta-analysis by Diao et al., including 39 studies, reported pooled estimates of malnutrition in children with CHD at 27.4% for underweight, 24.4% for stunting, and 24.8% for wasting, using the same WHO definitions employed in our study [8]. Significant heterogeneity between continents was observed, with the highest prevalence reported in Africa (42.5% for underweight, 45.4% for stunting, and 31.5% for wasting) and the lowest in North America (16.2% for underweight and 14.8% for wasting) and Oceania (20.5% for stunting) [8]. Moreover, based on the malnutrition criteria provided by the ASPEN consensus statement, 33.8% of the children in our study were malnourished, with the children younger than 24 months showing significantly higher odds of malnutrition compared to the older children. The higher prevalence in younger children, particularly in infants, was documented in previous studies, and this might be explained by the greater metabolic demands, increased energy expenditure, delayed feeding milestones, and inadequate nutrient intake during this critical period of growth [30,31,32,33]. In addition, it is noteworthy to mention that the CHD type as well as its severity and complexity can have an important effect on the end-organ perfusion and oxygenation. In particular, structural left-sided obstructions or defects can lead to systemic hypoperfusion, thus limiting oxygen supply and nutrient delivery to the systemic organs [34]. Our study showed that the frequency of malnutrition varied according to the underlying CHD type and its associated hemodynamic effects. Notably, our study revealed that the most common CHDs were VSD and TOF. Interestingly, children with VSD had higher odds of being malnourished than the subjects with TOF. Similar findings were reported by Ross et al. in their analysis of data from the Society of Thoracic Surgeons Database [28]. In fact, although VSD is an acyanotic CHD, the left-to-right shunting leads to excessive pulmonary blood flow, further increasing the energy needs, which, in turn, exacerbate the risk of malnutrition. In contrast, while TOF is a cyanotic CHD, characterized by chronic hypoxemia, children with TOF are comparatively protected from pulmonary blood flow and typically undergo corrective cardiac surgeries at an early age, therefore preventing the effect of chronic cyanosis on malnutrition [28,35].

The impact of malnutrition on postoperative outcomes was a key focus of our study, and our results provided important insights into the effects of different anthropometric indicators. While several previous studies have examined the associations between nutritional status and postoperative mortality in children with CHDs [13,14,15], the low in-hospital mortality rate (1.4%) in our study limited the ability to assess the relationship between malnutrition and mortality. Further studies with larger sample sizes or multicenter collaborations might allow for more comprehensive analysis. Contrary to previous studies [14,15,36], our findings suggest that malnutrition, stunting, wasting, or underweight did not have any substantial impact on the risk of postoperative infection. This finding is noteworthy given the well-established role of malnutrition in compromising immune function [26,27,28]. One likely explanation is that rigorous perioperative care including strict infection control measures and appropriate prophylactic antibiotic use might have mitigated the impact of malnutrition on the postoperative infection rate.

A lower WAZ and, more specifically, underweight, defined as WAZ < −2, were associated with a longer PICU stay. A longer hospital stay was also observed with a decreasing WAZ. Several previous studies support these observations [14,15,37,38,39]. Although a decrease in the HAZ was linked to a longer PICU stay, the binary classification of stunting (HAZ < −2) did not reach statistical significance in predicting adverse outcomes. In line with our findings, Ross et al. found that both a lower WAZ and HAZ were significantly associated with a longer PICU stay among infants and children with CHDs undergoing cardiac surgery at Seattle’s Children hospital between 2006 and 2015 [14]. Overall, underweight status appeared to have a more pronounced association with the length of hospital stay and PICU stay, compared to stunting and wasting. This result is consistent with the findings reported by Wittenberg et al. in their study on the burden of malnutrition on the early postoperative outcomes after VSD closure in children under 5 years of age [15]. According to the WHO, a low weight for age is indicative of stunting, wasting, or both, thereby reflecting both acute and chronic malnutrition. In contrast, a low height for age is the result of chronic or recurrent undernutrition, while wasting often indicates recent weight loss or acute undernutrition [10,40]. Therefore, our results might be partially understood in this context. Since WAZ captures both acute and long-term nutritional deficits, it might be more sensitive in identifying children at higher risk of complications. In contrast, the HAZ may not fully capture acute nutritional deficits that directly affect surgical recovery, and the WHZ, although indicative of acute malnutrition, might not detect the cumulative effect of long-term undernutrition. Furthermore, the challenges with obtaining accurate height or length measurements, especially in small or critically ill children, can introduce variability, potentially underestimating the true impact of the HAZ and WAZ on clinical outcomes [15]. It is worth noting that while the WAZ provides a useful, albeit rough indicator of the nutritional status, it also has significant limitations, particularly when measured at a single point. This measurement can be affected by acute fluid shifts, capillary leaks, and edema [13]; therefore, serial measurements or the inclusion of additional parameters such as the mid-upper arm circumference might provide a better estimation of malnutrition and its impact on clinical outcomes [21].

Furthermore, when we applied the ASPEN criteria to classify malnutrition—a more standardized and comprehensive assessment of nutritional status by integrating various anthropometric measures and clinical parameters [21]—we documented a significant association with a longer PICU stay in both logistic regression and the generalized linear models. This suggests that, by using ASPEN criteria, we might have been able to uncover some of the associations that were obscured when relying solely on individual nutritional indicators, effectively capturing the multifaceted nature of both acute and chronic malnutrition.

While the lower BMI z-score was associated with prolonged mechanical ventilation, other malnutrition indicators did not show this association. BMI is commonly used to assess the nutritional status in adults [41,42], and numerous studies have established a link between a lower BMI and adverse postoperative outcomes following cardiac surgeries [43,44,45]. However, the application of BMI-for-age z-scores in pediatric populations, particularly in children with CHDs, remains underexplored. A study published in 2017 by Bechard et al., using the BMI z-score as an indicator for nutritional status, demonstrated that children with a BMI Z-score < −2 had a higher risk of mortality and infections and fewer ventilator-free days [46]. This finding underscores the importance of including BMI z-scores in nutritional assessments, particularly in older children, where this metric may more accurately reflect body composition and its impact on clinical outcomes. Nevertheless, the use of BMI as a stand-alone measure has been increasingly questioned as it does not differentiate between fat mass (adipose tissue) or fat-free mass (lean body mass) [47].

The main strength of this study is that it offers comprehensive data on the epidemiology of malnutrition and its impact on postoperative outcomes among infants and children with CHDs in Lebanon. The use of standardized nutritional criteria (ASPEN), multiple standardized anthropometric indicators and well-defined clinical outcomes offers a robust assessment of malnutrition in a high-risk population and enhances the reliability of our findings. However, there are some limitations to consider. This study reported a single-center experience (AUBMC), which may limit the generalizability of the results; however, AUBMC hosts the Children’s Heart Center, which is a major referral center caring for children with CHDs in Lebanon. Moreover, the study included 139 subjects which, while sufficient for the analysis, may not fully represent the broader population of children with congenital heart disease (CHD). A larger sample size would provide more robust findings and allow for subgroup analyses. Additionally, one limitation is the lack of assessment of serum biochemical markers such as pre-albumin or albumin, BNP, transferrin, and additional nutritional status indicators such as body composition measures (triceps skinfold z-score and mid-arm circumference) due to its retrospective nature [37,48,49,50]. We did not account for other potential confounding factors of adverse outcomes such as pre-existing cardiac dysfunction or heart failure, aortic cross-clamp duration, and cardiopulmonary bypass duration, which have been proven to have an association between the z-score/BMI and length of ICU stay and mechanical ventilation period [13,51]. One important limitation of our study is that we did not account for the specific nutritional interventions provided, nor did we assess the actual caloric intake of the patients during the pre- and postoperative period.

## 5. Conclusions

This study underscores the significant impact of malnutrition, particularly underweight, as assessed by the WAZ, on postoperative outcomes in children with CHDs. Implementing targeted nutritional support interventions in the perioperative period is crucial for improving recovery and avoiding complications in this high-risk population. Further research should prospectively track the nutritional status of infants and children with CHDs both pre- and postoperatively to better understand the impact of malnutrition on clinical outcomes. In addition, evaluating nutritional interventions, conducting multicenter, large-scale studies, and assessing the influence of socio-economic factors are also crucial. These efforts could inform policy recommendations and nutritional care protocols, therefore optimizing surgical outcomes and ultimately reducing the burden of postoperative complications in this vulnerable population.

## Figures and Tables

**Table 1:** Demographic and clinical characteristics by nutritional status (according to ASPEN criteria).

	Normal Nutritional Status, n (%) *n* = 92	Malnourished, n (%) *n* = 47	*p*-Value
**Age groups**			**0.001**
<24 months	52 (56.5)	40 (85.1)	
≥24 months	40 (43.5)	7 (14.9)	
**Gender**			0.326
Male	55 (59.8)	24 (51.1)	
Female	37 (40.2)	23 (48.9)	
**Type of CHD**			0.218
Acyanotic	35 (38.0)	23 (48.9)	
Cyanotic	57 (62.0)	24 (51.1)	
**RACHS-1 categories**			0.536
1 to 3	81 (88.0)	43 (91.5)	
4 to 6	11 (12.0)	4 (8.5)	
**Pulmonary hypertension**	13 (14.1)	11 (23.4)	0.171

Pearson’s Chi-Square test was used (no expected count less than 5). The bold values refer to the significant factors that have a *p*-value < 0.05. ASPEN: American Society for Parenteral and Enteral Nutrition; CHD: congenital heart disease; RACHS-1: Risk Adjustment for Congenital Heart Surgery.

**Table 2:** Comparison of clinical outcomes between malnourished patients and those with normal nutritional status.

	Overall (*n* = 139)		
	Normal Nutritional Status (*n* = 92), n (%)	Malnourished (*n* = 47), n (%)	*p*-Value
**In-hospital mortality**	2 (2.2)	0 (0.0)	0.549 *
**Infection**	29 (31.5)	16 (32.7)	0.764
**Length of hospital stay, median (IQR)**	9.0 (7.0-14.0)	8.0 (7.0-15.0)	0.725
**Length of hospital stay**			0.643
≤7 days	24 (26.1)	14 (29.8))	
>7 days	68 (73.9)	33 (70.2)	
**Length of PICU stay, median (IQR)**	2.0 (2.0-3.0)	3.0 (2.0-7.0)	**0.004**
**Duration of PICU stay**			**0.008**
≤3 days	71 (77.2)	28 (55.3)	
>3 days	21 (22.8)	21 (44.7)	
**Inotropes use**	51 (55.4)	25 (53.2)	0.802
**Mechanical ventilation time**			
<48 h	79 (85.9)	37 (78.7)	0.672
≥48 h	13 (14.1)	10 (21.3)	
**Mechanical ventilation time, median (IQR)**	5.0 (3.0-16.5)	9.0 (4.0-44.0)	**0.017**

Pearson’s Chi-Square test was used (no expected counts less than 5). * Fisher’s exact test was used when expected count was less than 5. Mann–Whitney U test was used to compare non-normally distributed continuous variables. The bold values refer to the significant results that have a *p*-value < 0.05. IQR: interquartile range; PICU: pediatric intensive care unit.

## Data Availability

The data are available upon request to the corresponding author due to privacy and ethical restrictions.

## Supplementary Materials

The following supporting information can be downloaded at https://www.mdpi.com/article/10.3390/children12060705/s1, Table S1. Demographic and clinical characteristics (overall and by age group). Table S2. Outcomes by nutritional status, stratified by age group. Table S3. Median (interquartile range) of hospital stay, PICU stay, and mechanical ventilation time by stunting, wasting, and underweight. Table S4. Correlation analysis of anthropometric indicators and postoperative outcomes. Table S5. Generalized linear models (GLMs) assessing the relationship between malnutrition indicators (both categorical and continuous) and clinical outcomes (length of hospital stay, duration of PICU stay, and mechanical ventilation time). Figure S1. The distribution of anthropometric indicators by age group. Figure S2. Distribution of the study population by measures of malnutrition (wasting, stunting, and underweight).
