# Supplementary material for: The Association Between Preoperative Malnutrition and Early Postoperative Outcomes in Children with Congenital Heart Disease: A 2-Year Retrospective Study at a Lebanese Tertiary Medical Center
Source: Children (Basel). 2025 May 29;12(6):705. doi: 10.3390/children12060705 (PMC12191067; doi:10.3390/children12060705)
Supplement: Supplementary file 1 [file children-12-00705-s001.zip › children-3558160-supplementary.pdf]

Supplementary Materials

Table S1. Demographic and clinical characteristics (overall and by age group).

	Total, n (%) N=139	<24 months, n (%) N=92	≥24 months, n (%) N=47	p-value
**Gender**				0.408
Male	79 (56.6)	50 (54.3)	29 (61.7)	
Female	60 (43.2)	42 (45.7)	18 (38.3)	
**Type of CHD**				0.385
Acyanotic	58 (47.7)	36 (39.1)	22 (46.8)	
Cyanotic	81 (58.3)	58 (60.9)	25 (53.2)	
**RACHS-1 categories**				0.536
1 to 3	124 (89.2)	81 (88.0)	43 (91.5)	
4 to 6	15 (10.8)	11 (12.0)	4 (8.5)	
**Pulmonary hypertension**	24 (17.3)	20 (21.7)	4 (8.5)	0.051
**Malnutrition (ASPEN)**				
No malnutrition	65 (46.8)	34 (37.0)	31 (66.0)	Ref
Mild	27 (19.4)	18 (19.6)	9 (19.1)	0.209
Moderate	16 (11.5)	13 (14.1)	3 (6.4)	**0.045**
Severe	31 (22.3)	27 (29.3)	4 (8.5)	**0.002**

Pearson's Chi-Square test was used (no expected count less than 5).

The bold values refer to the significant factors that have a p-value <0.05.

CHD: Congenital heart disease; RACHS-1: Risk Adjustment for Congenital Heart Surgery; ASPEN: American Society for Parenteral and Enteral Nutrition.

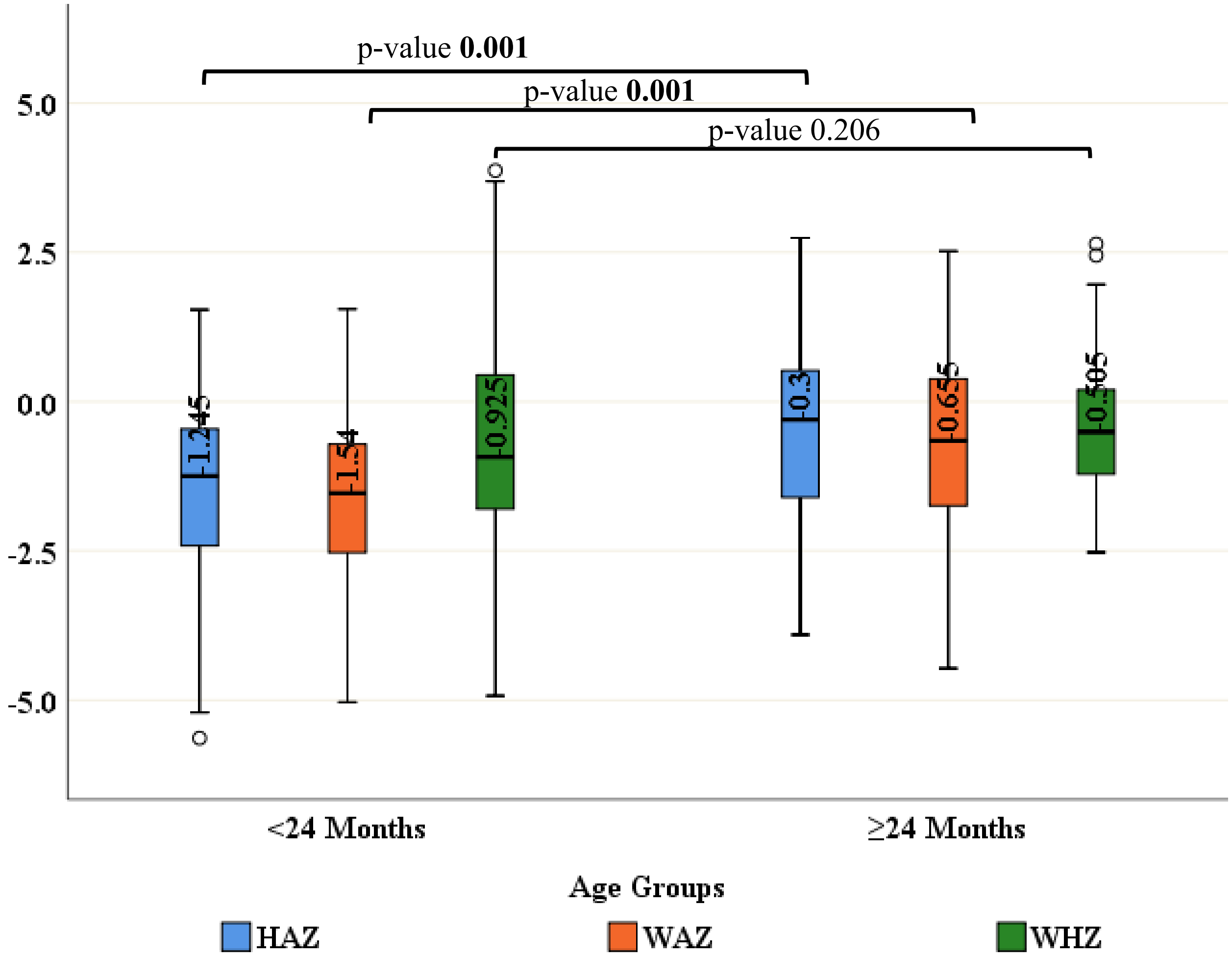

Figure S1. The distribution of anthropometric indicators by age groups.
Boxplot showing the distribution of HAZ, WAZ and WHZ across the two age groups. Independent samples t-test was performed to compare means between groups.
WAZ: weight-for-age z-score, HAZ: Height for age z-score, WHZ: weight-for-height z-score.

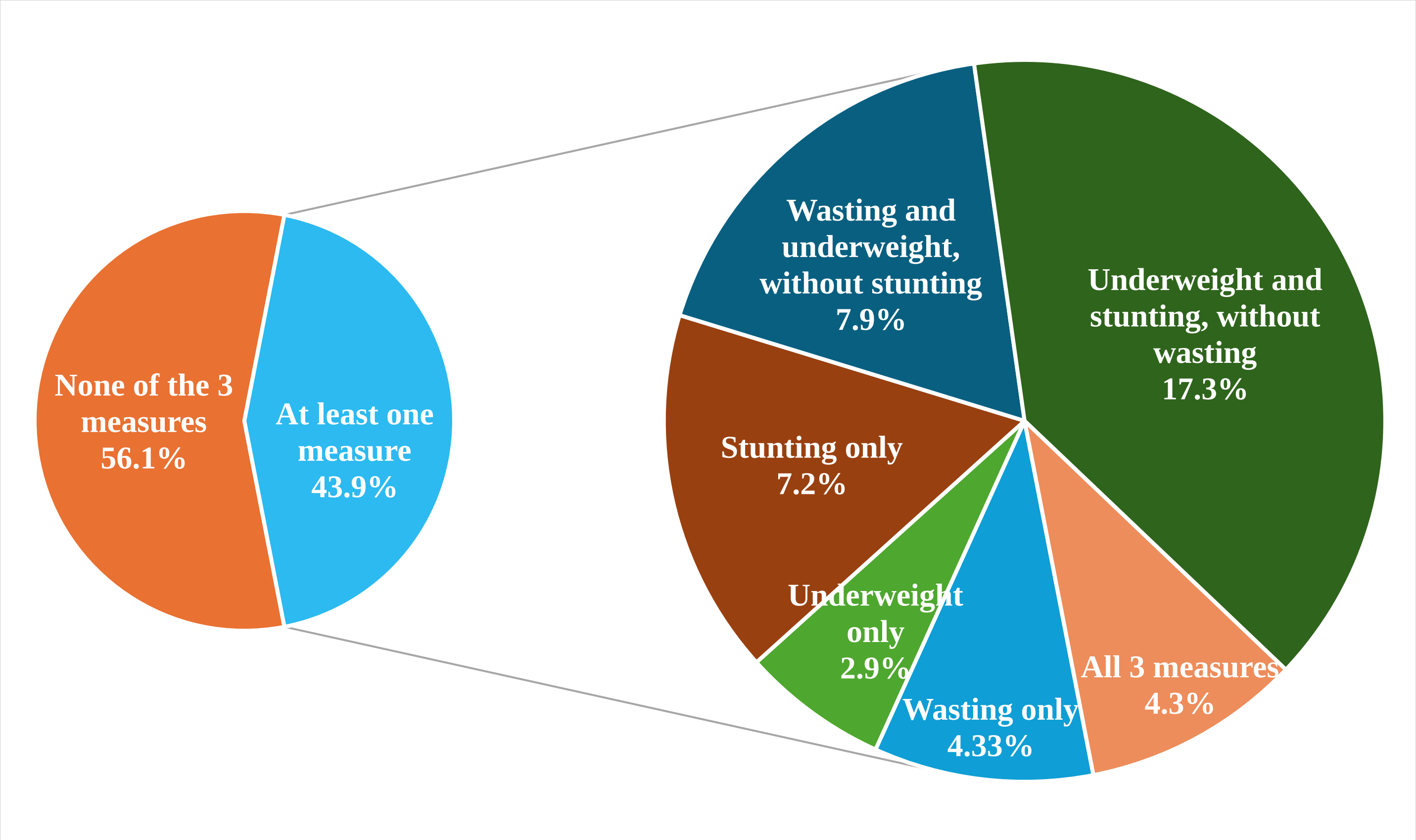

Figure S2. Distribution of the study population by measures of malnutrition (wasting, stunting and underweight).

The first pie chart shows the percentage of patients meeting criteria for at least one measure of malnutrition versus those who met criteria none, while the second pie chart illustrates the proportion of patients meeting one, two, or all three malnutrition criteria

Table S2. Outcomes by nutritional status, stratified by age groups.

	< 24 months (N=87)			≥ 24 months (N=52)		
	Normal nutritional status (N=52) n (%)	Malnourished (N=40), n (%)	p-value	Normal nutritional status (N=40) n (%)	Malnourished (N=7), n (%)	p-value
**In-hospital mortality**	1 (2.0)	0 (0.0)	1.000*	1 (2.5)	0 (0.0)	1.000*
**Infection occurrence**	24 (49.0)	13 (34.2)	0.208	4 (10.0)	2 (28.6)	0.214*
**Length of hospital stay, median (IQR)**	10.0 (8.0-14.0)	8.0 (7.0-16.0)	0.573	9.0 (7.0-14.0)	9.0 (6.0-11.0)	0.809
**Length of hospital stay**			0.230			1.000*
≤ 7 days	10 (19.2)	12 (30.0)		14 (34.1)	4 (42.9)	
> 7 days	42 (80.8)	28 (70.0)		27 (65.9)	7 (63.6)	
**Length of PICU stay, median (IQR)**	2.0 (2.0-5.5)	3.0 (2.0-9.0)	0.171	2.0 (1.0-2.0)	4.0 (2.5-5.0)	**0.021**
**Duration of PICU stay**			0.334			**0.011***
≤ 3 days	35 (67.3))	23 (57.5)		36 (90.0)	3 (42.9)	
>3 days	17 (32.7)	17 (42.5)		4 (10.0)	4 (57.1))	
**Inotropes use**	33 (63.5)	21 (52.5)	0.290	18 (45.0)	4 (57.1)	0.690*
**Mechanical ventilation time**			0.830			1.000*
**< 48 hours**	40 (76.9)	30 (70.0)		39 (97.5)	7 (100)	
**≥ 48 hours**	12 (23.1)	10 (25.0)		1 (2.5)	0 (0.0)	
**Mechanical ventilation time, median (IQR)**	7.0 (3.0-38.0)	14.5 (4.0-46.0)	0.265	4.0 (2.0-6.0)	6.0 (3.5-10.0)	0.372

Pearson's Chi-Square test was used (no expected count less than 5).
*Fisher's exact test was used when expected count was less than 5.
Mann-Whitney U test was used to compare non normally distributed continuous variables
The bold values refer to the significant results that have a p-value <0.05.
IQR: Interquartile range; PICU: Pediatric intensive care unit.

Table S3. Median (Interquartile Range) of hospital Stay, PICU stay, and mechanical ventilation time by stunting, wasting and underweight.

	Median (IQR)					
	**No stunting (N=99)**	**Stunting (N=40)**	**Not underweight (N=94)**	**Underweight (N=45)**	**No wasting (N=115)**	**Wasting (N=23)**
**Length of hospital stay (in days)**	9.0 (7.0-14.0)	9.0 (7.0-15.5)	9.0 (7.0-14.0)	9.0 (7.0-16.0)	9.0 (7.0-14.5)	8.0 (7.0-13.5)
**Duration of PICU stay (in days)**	2.0 (2.0-4.0)	3.0 (2.0-5.50)	**2.0 (2.0-3.0)**	**3.0 (2.0-8.0)**	2.0 (2.0-4.0)	3.0 (2.0-8.5)
**Mechanical ventilation time (in hours)**	6.0 (3.0-19.0)	6.0 (3.0-23.0)	5.0 (3.0-19.0)	7.0 (3.0-44.0)	5.0 (3.0-19.5)	10.0 (4.5-20.5)

Values are presented as median (IQR). Differences between groups were not statistically significant, except for longer and more variable PICU stay observed in underweight patients (p-value = 0.02).

Table S4. Correlation analysis of anthropometric indicators and postoperative outcomes.

	**Mechanical ventilation time (hours)**	**Length of hospital stay (days)**	**Duration of PICU stay (days)**
**WAZ (N=139)**	ρ: -0.147; p-value: 0.083	ρ: -0.067; p-value: 0.433	**ρ: -0.291; p-value: 0.001**
**HAZ (N=139)**	ρ: -0.112; p-value: 0.187	ρ: -0.094; p-value: 0.273	**ρ: -0.229; p-value: 0.007**
**WHZ (N=138)**	ρ: -0.014; p-value: 0.873	ρ: -0.002; p-value: 0.979	ρ: -0.109; p-value: 0.204
**BMI-for-age z-score (N=47)**	ρ: 0.001; p-value: 0.994	ρ: -0.191; p-value: 0.198	ρ: -0.189; p-value: 0.202

Spearman's rank correlation coefficient (rho; ρ) was used to represent the strength of association;
WAZ: weight-for-age z-score; HAZ: height-for-age z-score; WHZ: weight-for-height z-score.
BMI-for-age z-score was used only in children aged 24 months or older.

Table S5. Generalized linear models (GLM) assessing the relationship between malnutrition indicators (both categorical and continuous) and clinical outcomes (length of hospital stay, duration of PICU stay, and mechanical ventilation time.

	Length of hospital stay (days)	Duration of PICU stay (days)	Mechanical ventilation time (hours)
**Categorical variables**		B (95% CI); p-value	
**Stunting**	0.21 (-0.02, 0.43); 0.070	0.32 (-0.02, 0.66); 0.064	0.38 (-0.13, 0.88); 0.141
**Wasting**	0.10 (-0.17, 0.37); 0.479	0.13 (-0.21, 0.53); 0.529	-0.29 (-0.86, 0.27); 0.313
**Underweight**	0.21 (-0.01, 0.42); 0.062	**0.55 (0.22, 0.87); 0.001**	0.44 (-0.3, 0.91); 0.067
**Malnutrition**	0.12 (-010, 0.34); 0.269	**0.36 (0.04, 0.68); 0.027**	0.42 (-0.06, 0.89); 0.085
**Continuous variables**		B (95% CI); p-value	
**Weight-for-age z-score**	**-0.07 (-0.14, -0.01); 0.036**	**-0.15 (-0.25, -0.05); 0.003**	-0.11 (-0.25, 0.03), 0.123
**Height-for-age z-score**	-0.06 (-0.12, 0.01); 0.080	**-0.11 (-0.19, -0.02); 0.015**	-0.11 (-0.23, 0.02); 0.091
**Weight-for-height z-score**	-0.4 (-0.11, 0.03_; 0.321	-0.03 (-0.13, 0.07); 0.567	0.08 (-0.07, 0.22); 0.306
**BMI-for-age z-score**	-0.11 (-0.24, 0.03); 0.112	**-0.21 (-0.36, -0.06); 0.007**	**-0.38 (-0.64, -0.13); 0.003**

The regression coefficients (B), 95% confidence intervals (CI), and p-values are reported for each malnutrition indicator.
PICU: Pediatric intensive care unit; BMI: Body mass index.
